# Ortho- and retronasal olfactory performance in rhinosurgical procedures: a longitudinal comparative study

**DOI:** 10.1007/s00405-020-06300-4

**Published:** 2020-08-19

**Authors:** Gerold Besser, David T. Liu, Gunjan Sharma, Tina J. Bartosik, Sebastian Kaphle, Max Enßlin, Bertold Renner, Christian A. Mueller

**Affiliations:** 1 Department of Otorhinolaryngology and Head and Neck Surgery, Medical University of Vienna, Währinger Gürtel 18-20, 1090 Vienna, Austria; 2 Institute of Experimental and Clinical Pharmacology and Toxicology, Friedrich-Alexander-Universität Erlangen-Nürnberg, Erlangen, Germany; 3 Institute of Clinical Pharmacology, Medical Faculty Carl Gustav Carus, Technische Universität Dresden, Dresden, Germany

**Keywords:** Anosmia, Chronic rhinosinusitis, ETDQ-7, Sniffin’ Sticks, SNOT

## Abstract

**Purpose:**

Testing olfaction should be an integral part of a clinical work-up in rhinosurgical procedures. Importantly, intact olfactory experience also includes retronasally perceived odors (retronasal olfaction). This study aimed at comprehensively assessing olfaction in patients undergoing rhinosurgical procedures in a comparative manner and evaluating relations to patient-reported outcome measurements (PROMs).

**Methods:**

Each nostril odor threshold and discrimination, and birhinal identification were tested using Sniffin’ Sticks in 14 subjects assigned for septoplasty (SP), 21 for septorhinoplasty (SRP), and 30 for endoscopic sinus surgery (ESS). The 27-Candy-Smell-Test was used to quantify retronasal abilities. Tests were repeated 3 months after surgery.

**Results:**

Olfactory dysfunction was preoperatively present in 21% of SP, in 47.6% of SRP, and in 80% of ESS patients. Odor threshold side differences were most frequently found in SRP. Frequently, SRP and ESS patients showed severely impaired retronasal olfaction. Half of included subjects re-visited after 3 months, but olfactory function did not improve overall and rarely on an individual basis to a meaningful extent. Subjective ratings on nasal patency and PROMs were not associated with olfaction nor with changes in olfactory scores.

**Conclusion:**

Olfactory function can decisively be impaired a priori not only in patients awaiting sinus surgery, but also in those assigned for functional septorhinoplasty. This impairment may not improve in the short term, which has to be taken into account in patient counseling. This study adds to the current literature on olfaction in rhinosurgery with the extension of retronasal testing.

## Introduction

Surgical procedures on or in the nose are performed daily and globally for several reasons. Beside cosmetic aspects, disturbances in nasal patency most commonly drive patients to undergo septoplasty (SP) or septorhinoplasty (SRP). Endoscopic sinus surgeries (ESS) also focus on other symptoms, such as pressure, rhinorrhea, and smell loss. In fact, olfactory dysfunction is a cardinal symptom and diagnosing criterion in rhinosinusitis, which, in its chronic state (CRS), often needs ESS [1]. However, in nasal surgeries other than ESS, the sense of smell is often neglected. Surgeons have to keep in mind though that the olfactory epithelium is embedded within the nose [in the olfactory cleft (OC) but also beyond this anatomic region [2]] and presumably, e.g., manipulation or scarring potentially influences this delicate area. Moreover, also changes in nasal anatomy may influence odorant transportation to the olfactory region.

Several previous studies assessed olfactory function in patients undergoing septoplasty (for a recent study and overview see [3]), fewer in septorhinoplasty [4–6]. All of these studies utilized available orthonasal olfactory tests and few of these in a lateralized manner (i.e., testing each nostril separately). But noteworthy, unilateral-based olfactory testing may reveal relevant side differences in up to 20% [7].

In regard to CRS, Kohli et al. summarized data and found olfactory measurements to improve following ESS, but concluded research is needed across olfactory dimensions [8]. Indeed, olfaction is more than the ability to identify an odorant but also to detect odorants at low concentrations, to discriminate and to memorize/recall odorants. Furthermore, the olfactory epithelium is also reached retronasally by odorants coming from the mouth through the pharynx (retronasal route). This ability of retronasal olfaction greatly contributes to flavor perception and, in consequence, helps us to enjoy dishes the way we do [9]. Since this back route can be affected differently from the orthonasal route (as shown in patients with nasal polyps and by mechanical obstruction of the anterior OC [10, 11]), testing retronasal olfactory function in rhinosurgical procedures may reveal critical information—possibly as an outcome predictor. To the best of our knowledge, retronasal olfaction has not been investigated in the course of rhinological surgeries in a comparative manner.

This longitudinal study, therefore, aimed at (i) fully assessing ortho- and retronasal olfactory performance prior to various rhinosurgical procedures, (ii) detecting changes of olfactory function with surgeries, and (iii) evaluating relations to disease-specific patient-reported outcome measurements (PROMs).

## Materials and methods

### Patients

**Fig. 1:**
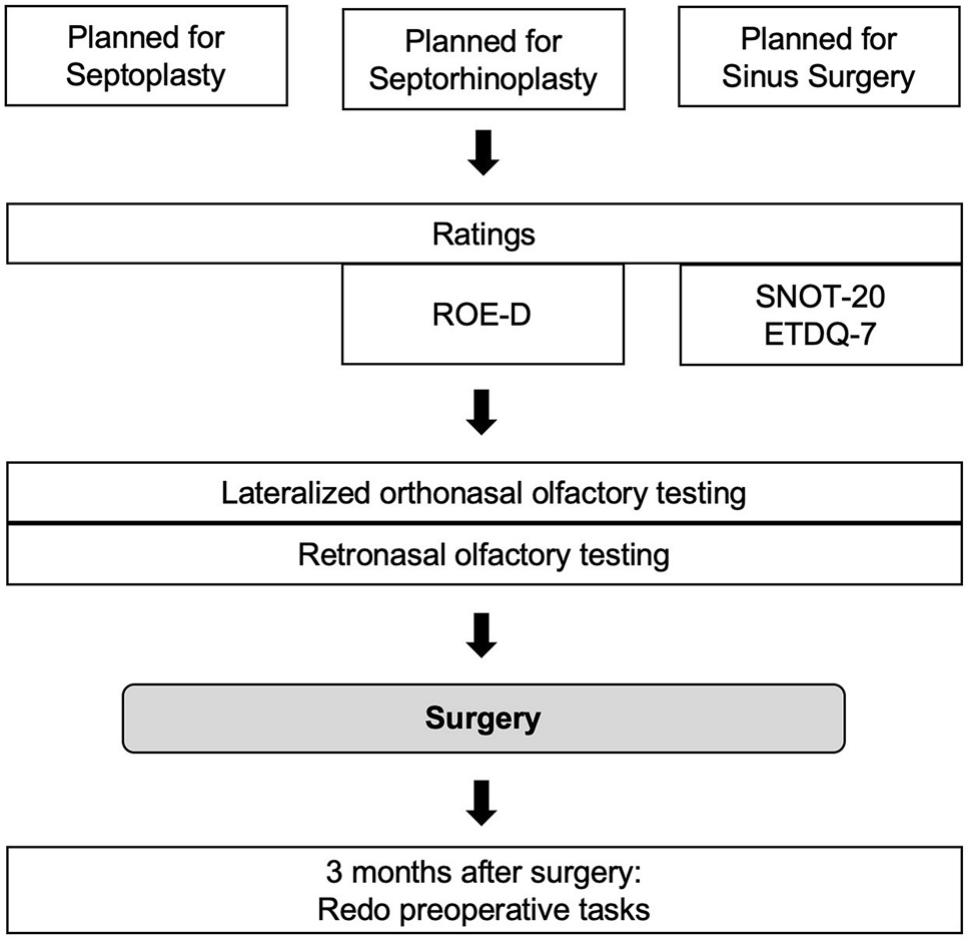
Study profile. *ETDQ-7* Eustachian tube dysfunction score, *ROE-D* German version of the rhinoplasty outcome evaluation questionnaire, *SNOT-20* sino-nasal outcome questionnaire

Sixty-five patients, 26 females and 39 males, with a mean age (mean ± standard deviation (SD)) of 38.3 ± 12.3, range 18–64 years, were prospectively recruited prior to rhinosurgical procedures. Patients, whose surgeries were canceled, were excluded. Fourteen participants underwent SP, 21 SRP, and 30 ESS. Patients’ characteristics are shown in Table 1. Olfactory testing (see below) took place on the day before surgery, sometimes, however, earlier due to postponed surgeries (2.0 ± 3.0 mean days before surgery). Of those assigned for ESS, 19 were classified as patients with CRS with nasal polyps (CRSwNP) and 11 as CRS without nasal polyps (CRSsNP) by means of intraoperative findings (i.e., surgeon reports polyps) and/or histological findings (i.e., polyp tissue ± eosinophilia). Postoperative olfactory function was planned to be tested 3 months after surgery. Most patients that did not re-visit were not reached or stated not to be willing to re-visit due to private issues. All tests took place between October 2018 and February 2020. For study profile, see Fig. 1.

**Table 1:** Demographics and clinical characteristics at baseline

	SP *n* = 14	SRP *n* = 21	ESS *n* = 30
Age	37.8 ± 9.4	37.6 ± 22.3	42.2 ± 13.4
Gender (*n*)	Female 3, male 11	Female 13, male 8	Female 10, male 20
BMI (kg/m^2^)	24.7 ± 4.3	25.9 ± 5.7	27.7 ± 4.8
Normosmic	11 (78.6%)	11 (52.3%)	6 (20%)
Hyposmic	3 (21.4%)	10 (47.6%)	18 (60%)
Anosmic	0	0	6 (20%)

*ESS* Endoscopic Sinus Surgery, *SP* Septoplasty, *SRP* Septorhinoplasty; in brackets: percentages of, e.g., normosmics within the group of SP

### Olfactory tests

Orthonasal olfactory abilities were assessed using the Sniffin’ Sticks test battery (Burghart GmbH, Wedel, Germany). Odor threshold (16 dilution steps) and odor discrimination (16 triplets) were tested for each nostril separately, while odor identification (16-item) was tested birhinally. Administration is described in detail elsewhere including normative data sets [12–16]. The score of three subtest was summed for each side separately (TDI left and right) with the best-performing nostril being used to categorize anosmia (16 or less), hyposmia (more than 16, less than 30.75) and normosmia (equal or above 30.75) [16].

Retronasal olfactory function was tested using the Candy-Smell-Test (CST) [17–19]. Validated in a 23-item version [17], in this study, we applied a 27-item version (for aromas and distractors, please see [20]). Candies were placed on the tongue and the target aroma had to be named out of a list of four possible answers.

### Patient-reported outcome measurements and ratings

Questionnaires were used in its German versions. In patients assigned for ESS, we applied the Sino-Nasal-Outcome-Test (20 items, SNOT-20) [21] and the Eustachian Tube Dysfunction Questionnaire-7 (ETDQ-7) [22, 23]. Both have been shown to be valuable in quantifying complaints in CRS [24, 25]. Patients awaiting septorhinoplasty were supplied with the Rhinoplasty Outcomes Evaluation questionnaire (ROE-D) [26]. This short 6-item questionnaire focuses on aesthetic aspects, but also asks for complaints in regard to nasal patency and can be used to measure surgical outcome [27].

All subjects rated their subjective nasal patency (SNP) for each side (from 0 mm left hand end = total block to 100 mm right hand end = excellent patency) on a visual analog scale. Subjects had to rate their abilities to smell (subjective assessment of smell, SAS) and perceive detailed flavors during eating and drinking (subjective assessment of flavor, SAF), like wine and herbs, on a ten-point scale (0 = no smell/flavor, 10 = excellent smell/flavor perception) prior to psychophysical testing. In case of SP and SRP, surgeons noted the most prominent side of septal deviations (septal deviation to the right or left).

### Statistical analysis

IBM SPSS 26.0 (IBM Corp., Armonk, NY, USA) and GraphPad Prism 8.2.0 (GraphPad Software, Inc., La Jolla, San Diego, CA, USA) were used for statistical analysis. Graphical visualization was performed using same GraphPrism. Normality of data was tested using Shapiro–Wilk test. Group differences were tested using (paired or unpaired) sample *t* test or Mann–Whitney test/Wilcoxon matched pairs test. Data are presented as mean and SD, as indicated. Correlational analyses were performed using the Pearson correlation coefficient (*r*). A *p* value of < 0.05 was required for statistical significance.

Individual changes in olfactory performance (pre- vs. postoperative) were interpreted as meaningful when reaching ± 2.5 points for odor threshold, ± 3 for odor discrimination and identification (hence ± 5.5 for TDI, see [28]) and ± 5 points for the CST [29].

## Results

### Preoperative olfactory test results

According to orthonasal TDI testing, of all participants, 28 were normosmic, 31 hyposmic, and 6 anosmic prior to surgery (see Table 1 for olfactory categories per surgical group). Mean CST scores were lowest in ESS subjects, followed by subjects assigned for SRP. Ortho- and retronasal test results correlated strongly significantly (*r*_64_ = 0.736; *p* < 0.0001), similar to the previous results in non-surgical subjects [18]. By grouping, this was also the case in the SRP (*r*_21_ = 0.666; *p* = 0.001) and ESS (*r*_29_ = 0.847; *p* < 0.0001) group, but not in the SP group (*p* > 0.05).

Relevant odor threshold side differences (≥ 3 points, see [7]) were found in 17 cases. Data on septal deviation was available in 30 cases of SP and SRP patients (14 deviations to the left and 16 to the right). Looking at relevant threshold differences and septal deviations: in 6 cases, there was a relevant lower threshold score on the deviated side; however, in 4 cases, threshold scores were lower on the non-deviated side. Relevant TDI side differences (≥ 6 points, see [7]) were found in 15 cases (see Table 2 for details). Olfactory differences were found between CRSsNP and CRSwNP (see Table 3 for details).

**Table 2:** Olfactory test results at baseline

	Surgical procedure			Intergroup differences		
	SP	SRP	ESS	SP vs. SRP	SRP vs. ESS	SP vs. ESS
TDI s-d	3 (21.4%)	5 (23.8%)	7 (23.3%)	–	–	–
TDI (best nostril)	31.9 ± 4.3	30.8 ± 4.1	24.7 ± 8.0	*p* = 0.2314	***p = 0.0009***	***p = 0.0004***
Threshold s-d	4 (28.6%)	8 (38.1%)	5 (16.7%)	–	–	–
Threshold right	5.9 ± 2.8	5.8 ± 2.8	4.1 ± 2.7	*p* = 0.9319	***p = 0.0459***	***p = 0.0616***
Threshold left	6.1 ± 2.6	5.9 ± 2.3	3.5 ± 2.5	*p* = 0.8060	***p = 0.0017***	***p = 0.0054***
Discrimination right	11.4 ± 2.1	9.9 ± 2.8	8.6 ± 3.2	*p* = 0.0732	*p* = 0.1190	***p = 0.0012***
Discrimination left	11.7 ± 1.9	11.0 ± 2.6	8.6 ± 2.7	*p* = 0.3898	***p = 0.0025***	***p = 0.0001***
Identification	12.5 ± 2.0	12.5 ± 1.7	10.4 ± 4.4	*p* = 0.9716	*p* = 0.1832	*p* = 0.2675
Candy-Smell-Test	19.4 ± 4.0	17.3 ± 5.2	15.2 ± 5.7	*p* = 0.2324	*p* = 0.1827	***p = 0.0121***

*ESS* Endoscopic Sinus Surgery, *s-d.* side difference, *SP* Septoplasty, *SRP* Septorhinoplasty, *TDI* summed score of odor threshold, discrimination, and identification; significant differences are in bold; in brackets: percentages of, e.g., subjects with relevant side differences within the group of SP

**Table 3:** Baseline differences of patients with and without nasal polyps

	CRSwNP	CRSsNP	*p* value
TDI	21.7 ± 9.3	29.8 ± 3.7	***p = 0.0012***
Candy-Smell-Test	13.4 ± 6.4	18.0 ± 3.4	***p = 0.0172***
SNOT	43.2 ± 15.8	32.4 ± 8.3	*p* = 0.0679
ETDQ-7	15.9 ± 7.5	11.4 ± 3.6	*p* = 0.0574

*CRSw/sNP* chronic rhinosinusitis with or without nasal polyps; significant differences are in bold

### Postoperative olfactory test results

Thirty-four patients (6 SP = 42.9%/10 SRP = 47.6%/18 ESS = 60.0%) re-visited 106.0 ± 12.8 days postoperatively for a second olfactory test cycle. Left and right TDI and CST scores did not change significantly pre- vs. postoperatively in overall subjects, nor for each procedure separately (all *p* > 0.151). Looking at orthonasal scores for each nostril neither threshold nor discrimination scores improved significantly 3 months after SP, SRP, or ESS (all *p* > 0.115). On an individual basis, best -performing nostril TDI score improved to a meaningful extent in nine cases (2 SP = 33.3%/1 SRP = 10.0%/6 ESS = 33.3%) and decreased in three cases (0 SP/1 SRP = 10.0%/2 ESS = 11.1%). CST scores improved to a meaningful extend in four cases (0 SP/2 SRP = 20.0%/2 ESS = 11.1%) and in 8 cases; however, it decreased (2 SP = 33.3%/1 SRP = 10.0%/5 ESS = 27.8%).

### Ratings and PROMs

At baseline, SAS and best-performing nostril TDI correlated moderately significantly (*r*_65_ = 0.593; *p* < 0.0001), whereas SAF and CST did not quite (*p* = 0.055). Per grouping, however, only in ESS subjects accurately rated their orthonasal olfactory abilities as measured by TDI (*r*_30_ = 0.688; *p* < 0.0001). No significant correlations were seen for SNP and corresponding side threshold, nor for collected PROMs and olfactory test results (all *p* > 0.05). SNP side differences were most prominent in patients assigned for SRP (SP *p* = 0.010; SRP *p* < 0.0001; ESS *p* = 0.004). ETDQ-7 (14.2 ± 6.6) and SNOT-20 (39.2 ± 14.3) correlated significantly (*p* = 0.0162).

**Fig. 2:**
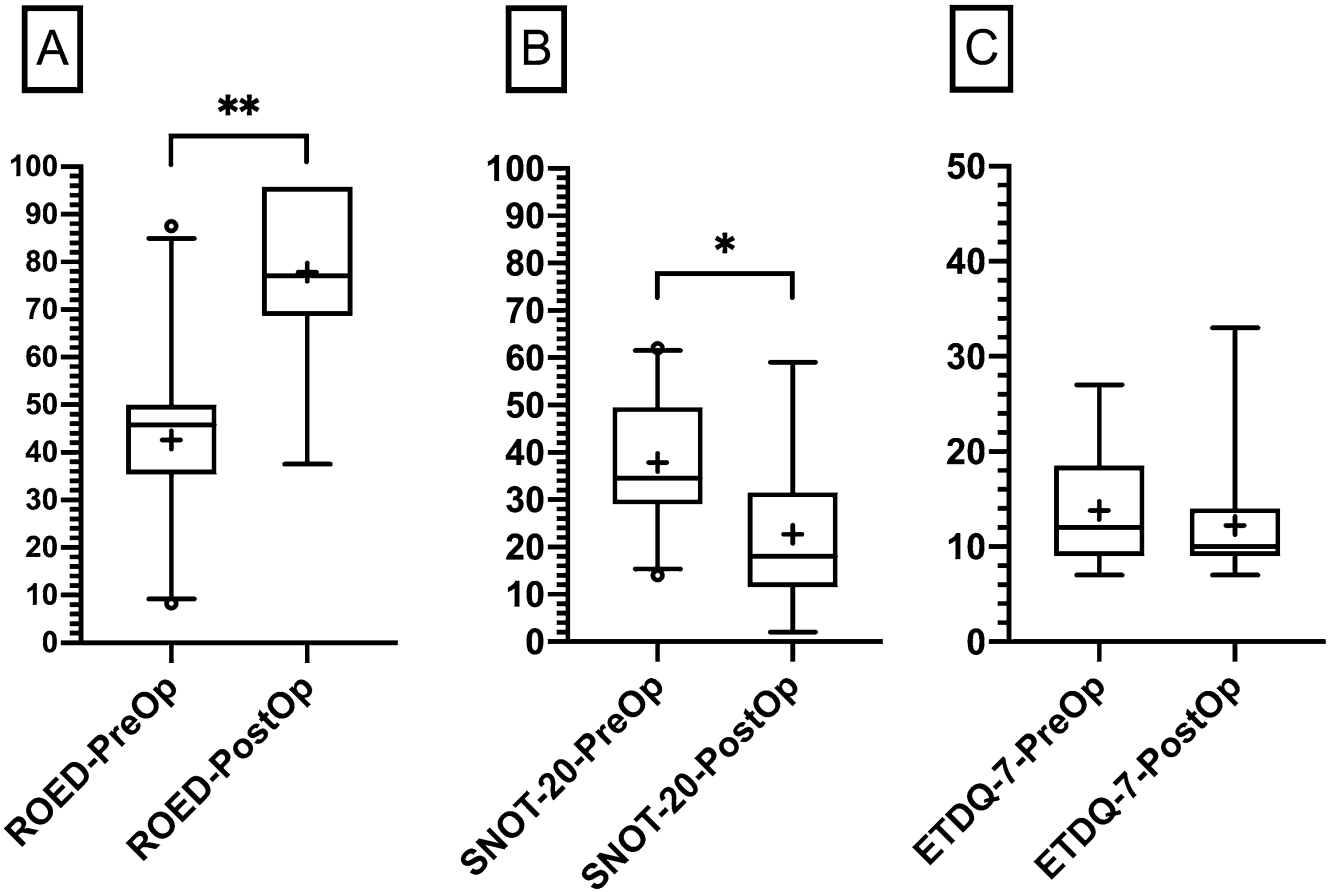
Box-and-whisker plots of patient-reported outcome measurements before and approximately 3 months after surgery. *ETDQ-7* Eustachian tube dysfunction score, *ROE-D* German version of the rhinoplasty outcome evaluation questionnaire, *SNOT-20* sino-nasal outcome questionnaire; medians (Q0.5; line), interquartile range (Q0.25, Q0.75; boxes); + indicating the mean scores; (*)*p* < 0.05, (**)*p* < 0.01; note that increasing ROE-D scores mirror improvements in contrast to the ETDQ-7 and SNOT

Summed SNP scores (left and right), ROE-D, and SNOT improved significantly 3 months after surgery (all *p* < 0.015), while ETDQ-7 scores did not differ significantly pre- vs. postoperatively. Table 4 shows results and *p* values, while Fig. 2 illustrates PROMs using box-and-whisker plots. Changes in SNOT-20 scores did not significantly correlate with changes in TDI (*r*_15_ = 0.430; *p* = 0.110). In 11 out of 15 collected SNOT scores in revisiting ESS subjects, the score improved to a meaningful extent (assuming a positive change in SNOT scores of ≥ 9 as meaningful in analogy to data published on the minimal clinically important difference (MCID) of the 22-item version [30]. CRSwNP had more relevant changes in SNOT scores (16.8 ± 15.7) compared to CRSsNP (7.8 ± 20.5); small sample size, however, limited statistical comparison.

**Table 4:** PROMs—results per group preoperatively vs. postoperatively

	Pre	Post	*p* value
SNP	77.3 ± 44.6	133.8 ± 34.4	***p < 0.0001***
SAS	5.5 ± 2.8	6.2 ± 2.3	*p* = 0.0923
SAF	6.5 ± 2.7	6.7 ± 2.1	*p* = 0.4800
SNOT	37.9 ± 13.4	22.7 ± 17.1	***p = 0.0140***
ETDQ-7	13.8 ± 3.9	11.1 ± 3.9	*p* = 0.2970
ROE-D	42.7 ± 17.0	77.9 ± 18.5	***p = 0.0030***

Significant differences are in bold

## Discussion

In this study, we put emphasize on comprehensive psychophysical olfactory testing in a prospective, unselected patient cohort assigned for the most commonly performed rhinosurgical procedures. Additionally, we investigated for relations of olfactory test results to patient-reported outcome measurements. These major findings emerged: olfactory dysfunction (OD) was most frequently present in the ESS group at baseline, but also very frequent in SRP patients (47.6%). Testing for multiple olfactory dimension, including retronasal testing, was able to unmask group differences, while sole orthonasal odor identification testing was not (see Table 1). Independently of type of surgery, olfactory function did not improve 3 months later and retronasal olfactory scores even worsened in eight subjects. Ratings and PROMs were mainly not associated with olfactory measurements, except for baseline ratings on smell in ESS patients. Although, subjective ratings on nasal patency, the ROE-D, and the SNOT improved significantly (hence, patients were quite satisfied with surgical outcome), these improvements did not correlate to a relevant extent with changes in olfaction.

Olfactory assessment in a pre-surgical setting is often reduced to sheer asking for complaints and/or fast screening tests, though both strategies can be misleading. Subjective ratings of olfactory capacity are inconsistent and may show significant correlations in patients with OD [19, 31]. On an individual basis, patients with OD of different etiologies do not rate their abilities consistently accurate. Moreover, in subjects naive to olfactory tests ratings may vary also depending on whether they are collected before or after testing [32]. Although subjective ratings can provide guidance in OD, also unnoticed anosmia or subjective dysfunction with normal test results are possible [33–35]. It has to be pointed out that most findings on self-ratings were collected in patients with various causes of OD, without a specific focus on sino-nasal disease. Data obtained in this study, therefore, add valuably to the literature specifically in regard to rhinosurgical patients. Guidelines exist for a clinical work-up in patients affected by various causes of OD, but a different approach in a pre-surgical setting (in patients with leading complaints other than olfaction) is not advocated. In general, subjective assessment is recommended to precede psychophysical screening testing. In case of abnormal screening results, it is recommended to perform further olfactory testing [35]. Our results show that comprehensive olfactory testing, including retronasal testing, may reveal important information for patient counseling, which may be overseen by sole orthonasal odor identification testing.

In contrast to SP, SRP in this cohort was performed via an open-approach with a transcolumellar incision. All procedures were performed due to functional, rather than aesthetic reasons, and frequently addressed the valve region and (in analogy to SP) septal deviations. Notable, olfactory function was found to be impaired in a significant number of patients assigned for SRP and olfactory side differences were surprisingly prominent in SRP patients (up to one-third), accompanied by prominent SNP side differences. Overall, we measured significant olfactory side differences in rhinological patients similar to previous authors in various OD patients [7]. Damm et al. described the anterior segment of the inferior nasal meatus to be of importance for odor thresholds [36]. It, therefore, may be hypothesized pronounced asymmetries in the nasal valve region as the underlying mechanism for side-difference findings. However, in the present study, there was a missing association of SNP (and side of septal deviation) and odor threshold scores. Furthermore, 3 months after SRP approximately the same percentage of subjects showed differences in odor threshold, although asymmetries should have been corrected by then (also since SNP improved significantly). Studies on this issue will be needed including objective tools like acoustic rhinometry.

Not many clinics perform extended olfactory tests, although numerous tests are commercially available and also self-administration has been proposed for Sniffin’ Sticks subtests saving personnel resources [37–39]. Retronasal tests have become more standardized, easy commercial accessibility, however, is still awaited [19, 40, 41]. Nevertheless, current knowledge on this topic encourages clinicians and researchers to test retronasal olfaction. This study demonstrated that retronasal olfaction can be severely impaired at baseline with low chances of improvement 3 months after surgery. Using standardized questionnaires, the previous authors showed retronasal olfaction to be predictive for quality of life [42]. Applied PROMs in this investigation, however, did not show associations with olfactory function. Further studies are needed on PROMs and their relations to olfactory function, especially for the ETDQ-7 and retronasal olfaction: the presence of nasal polyps affected retronasal olfaction in this cohort significantly and, therefore, future investigators may find associations of ear-related symptoms (hence retronasal symptoms) and retronasal olfaction.

This study certainly has some limitations. Only half of the subjects re-visited, and measurements were taken solely 3 months after surgery. This may add an unintended selection bias on comparative results of the re-visit test cycle. Participation was fairly time consuming given all tests and questionnaires; this has evidently affected compliance. Our group, however, attempted to collect broad psychophysical data to evaluate for prognostic factors. Future investigators may consider cutting down on tests ensuring participation compliance and implementing more time points. Furthermore, we did not apply standardized polyp size grading, which may have been able to elucidate associations to lateralized test scores. Noteworthy, gradings in septoplasty and septorhinoplasty need further standardization to be commonly applicable [43]. One strength to be pointed out: in regard to changes in olfactory function and PROMs, we applied MCID values whenever possible, aligning with recommendations [35, 44].

## Conclusion

Rhinological surgeons have to be aware of the sense of smell, especially its importance for flavor perception. Olfactory function, including retronasal olfaction, can decisively be impaired a priori not only in patients awaiting sinus surgery but also in those assigned for septorhinoplasty. Expectations on short-term improvement should be addressed with caution and a potential worsening of olfactory function needs to be a part in patient counseling. This study adds to the current literature on olfaction in rhinosurgery with the extension of retronasal testing.
